# Dynamic biomarkers and Cox regression with time-dependent covariate for mortality prediction in severe fever with thrombocytopenia syndrome

**DOI:** 10.1038/s41598-025-94416-0

**Published:** 2025-03-18

**Authors:** Hyun Ji Woo, Sang Taek Heo, Jeong Rae Yoo, Misun Kim, Jaeseong Oh, In-Gyu Bae, Sohyun Bae, Young-Ran Yoon, Jeong-Hwan Hwang, Miri Hyun, Hyun ah Kim, Sook In Jung, Ki Tae Kwon, Soyoon Hwang, Uh Jin Kim, Gaeun Kang, Young Jun Kim, Ji Hyun Yun, Tae-Eun Kim, Tae-Kyu Kwon, Min-Gul Kim

**Affiliations:** 1 Department of Healthcare Engineering, Graduate School, Jeonbuk National University, Jeonju, Republic of Korea; 2 Nanum Space Co., Ltd, Jeonju, Jeonbuk Republic of Korea; 3 Division of Infectious Diseases, Department of Internal Medicine, Jeju National University Hospital, Jeju National University School of Medicine, Jeju, Republic of Korea; 4 Department of Pharmacology, Jeju National University Hospital, Jeju National University School of Medicine, Jeju, Republic of Korea; 5 Division of Infectious Diseases, Department of Internal Medicine, Gyeongsang National University Hospital, Gyeongsang National University School of Medicine, Jinju, Republic of Korea; 6 Division of Infectious Diseases, Department of Internal Medicine, Kyungpook National University Hospital, Kyungpook National University School of Medicine, Daegu, Republic of Korea; 7 Department of Clinical Pharmacology, Kyungpook National University Hospital, Kyungpook National University School of Medicine, Daegu, Republic of Korea; 8 Division of Infectious Diseases, Department of Internal Medicine, Jeonbuk National University Hospital, Jeonbuk National University Medical School, Jeonju, Republic of Korea; 9 Division of Infectious Diseases, Department of Internal Medicine, Keimyung University Dongsan Hospital, Keimyung University School of Medicine, Daegu, Republic of Korea; 10 Division of Infectious Diseases, Department of Internal Medicine, Chonnam National University Hospital, Chonnam National University Medical School, Gwangju, Republic of Korea; 11 Division of Infectious Diseases, Department of Internal Medicine, Kyungpook National University Chilgok Hospital, Kyungpook National University School of Medicine, Daegu, Republic of Korea; 12 Division of Infectious Diseases, Department of Internal Medicine, Chonnam National University Hwasun Hospital, Chonnam National University Medical School, Gwangju, Republic of Korea; 13 Division of Clinical Pharmacology, Department of Pharmacology, Chonnam National University Hospital, Chonnam National University Medical School, Gwangju, Republic of Korea; 14 Division of Infectious Diseases, Department of Internal Medicine, Wonkwang University Hospital, Iksan, Republic of Korea; 15 Division of Infectious Diseases, Department of Internal Medicine, Konkuk University Medical Center, Konkuk University School of Medicine, Seoul, Republic of Korea; 16 Department of Clinical Pharmacology, Konkuk University Medical Center, Seoul, Republic of Korea; 17 Division of Biomedical Engineering, College of Engineering, Jeonbuk National University, Jeonju, Republic of Korea; 18 Department of Pharmacology, Jeonbuk National University Hospital, Jeonbuk National University Medical School, Jeonju, Republic of Korea

**Keywords:** Severe fever with thrombocytopenia syndrome, Time-Dependent covariate, Dynamic biomarkers, Mortality prediction, Infectious-disease diagnostics, Infection

## Abstract

Severe fever with thrombocytopenia syndrome (SFTS) is a fatal tick-borne infectious disease that lacks effective treatments. Dynamic analysis that reflects changes in the SFTS patient’s condition is needed. This study aimed to evaluate the time-dependent predictive performance of key biomarkers using a time-dependent Cox regression model. A retrospective multicenter cohort study was conducted on 440 SFTS patients hospitalized in South Korea between 2013 and 2024. Time-dependent Cox regression and time-dependent receiver operating characteristic (ROC) analyses were applied to assess the prognostic value of Blood Urea Nitrogen (BUN), Prothrombin Time (PT), and Activated Partial Thromboplastin Time (aPTT). Missing data were handled using multiple imputation. aPTT consistently demonstrated high predictive accuracy (AUC > 0.90) throughout the disease course, indicating its sustained role in coagulopathy. PT exhibited strong early-stage predictive power (AUC = 0.86 on day 2) but declined over time, reflecting its utility for early monitoring. BUN showed a progressive increase in predictive performance (AUC = 0.70 on day 2 to AUC = 0.78 on day 8), supporting its relevance in later stages of disease progression. Non-survivors exhibited significantly higher levels of BUN, PT, and aPTT compared to survivors. This study demonstrates the utility of time-dependent analysis for evaluating dynamic biomarker changes in SFTS patients. aPTT is a robust predictor throughout the disease course, while PT is valuable for early-stage assessment and BUN for later-stage management. These findings suggest the importance of integrating dynamic biomarker monitoring into clinical decision-making to improve prognosis in SFTS patients.

**Supplementary Information:**

The online version contains supplementary material available at 10.1038/s41598-025-94416-0.

## Introduction

Severe Fever with Thrombocytopenia Syndrome (SFTS) is an acute viral disease caused by the SFTS virus, primarily transmitted through tick bites, and is a zoonotic infection. Since its first report in China in 2009, SFTS has emerged as a severe viral disease characterized by high incidence and mortality, particularly in East Asia (China, Japan, Korea, and Vietnam)^1–4^. There is currently no available vaccine or antiviral treatment, and the disease progresses rapidly, with a mortality rate of approximately 20%, which is higher than that observed for other infectious diseases^5^. Early detection and prompt treatment are crucial. Therefore, there is an urgent need to develop clinical prediction models that can accurately assess the prognosis of SFTS and optimize patient management.

Time-dependent covariates frequently arise in biomedical research evaluating patient status. For instance, a patient’s serum creatinine level, which reflects renal function, may fluctuate during hospitalization. In contrast, time-fixed covariates are variables whose values remain unchanged throughout the study period.

Previous studies have identified demographic factors, comorbidities, and initial clinical status as significant factors influencing the prognosis of SFTS^6–9^. These studies mainly focus on assessing the association between early biomarker values, measured on the day of hospital admission, and survival risk, using the Cox proportional hazards model (Cox model), which is based on time-fixed covariates. Although the Cox model is useful for assessing risk at a specific point in time, it has the limitation of not reflecting the patient’s dynamically changing condition^10,11^.

In order to make informed decisions regarding the treatment and management of patients in actual clinical settings, more advanced analyses that dynamically reflect changes in a patient’s condition are needed^12^. In particular, for acute infectious diseases such as SFTS, clinical outcomes in the initial state can change rapidly over time, so predicting mortality based on this is highly likely to result in unreliable results.

Recently, considerable research has been conducted on time-dependent covariate models that overcome the limitations of existing time-fixed covariate-based models and effectively reflect the dynamic characteristics of data^13–15^. Unlike models that reflect only a specific point in time, time-dependent covariate models integrate longitudinal data and include it in the analysis, allowing the dynamic characteristics of the data to be reflected in the analysis. By dynamically analyzing the impact of changes in clinical status at a specific point in time on death, more accurate predictions and interpretations can be achieved compared to single-point analysis.

Therefore, this study aimed to analyze the factors influencing in-hospital mortality in SFTS patients using a time-dependent covariate model. The objective of this study is to identify dynamically changing risk factors, accurately assess the prognosis of SFTS patients, and provide a foundation for supporting clinical decision-making.

## Methods

### Study design and patient selection

This retrospective, multicenter cohort study was conducted nationwide in South Korea, utilizing data from ten tertiary referral hospitals with infectious diseases specialists. Patients with a confirmed diagnosis of SFTS who were hospitalized and treated between May 2013 and October 2024 were included. SFTS virus infection was confirmed by detecting viral RNA in patient serum during the acute phase of illness using real-time polymerase chain reaction (RT-PCR) or conventional reverse transcription PCR. Patients who were not admitted to the ward-namely, those evaluated only in the outpatient clinics or who left the hospital against medical advice-were excluded. Additionally, patients with no laboratory tests performed within one day of hospital admission were excluded, as baseline laboratory results were unavailable for the admission day or the following day.

The study protocol was approved by the Institutional Review Boards (IRBs) of each participating hospital, and the list of IRB approvals and numbers is provided in Supplementary Table 1. Due to the retrospective nature of the study and the use of anonymized patient data, the requirement for written informed consent was waived.

## Data collection

Demographic, clinical, and laboratory data were collected from the electronic medical records (EMRs) of each hospital. Laboratory data comprised test results obtained throughout the hospital stay. Key hematological and biochemical markers such as white blood cell (WBC) count, platelet count, aspartate aminotransferase (AST), alanine aminotransferase (ALT), blood urea nitrogen (BUN), creatinine, lactate dehydrogenase (LDH), albumin, C-reactive protein (CRP), prothrombin time (PT), and activated partial thromboplastin time (aPTT) were included. The primary outcome variable for this study was in-hospital mortality, defined as death occurring during the hospital stay.

### Statistical analysis

All statistical analyses were performed using R software version 4.4.2 (R Foundation for Statistical Computing, Vienna, Austria). Continuous variables were expressed as mean ± standard deviation or median (interquartile range) based on the data distribution, and categorical variables were presented as frequencies and percentages.

To assess the dynamic effect of biomarkers on mortality, a time-dependent Cox regression model was applied. The dataset for time-dependent Cox regression analysis was created using the tmerge function from the *survival* package. Each laboratory test result was categorized into time intervals based on the test date and converted into time-dependent covariates.

To handle missing laboratory test data, multiple imputation was performed using the *mice* (Multivariate Imputation by Chained Equations) package in R. Twenty imputed datasets were generated using predictive mean matching with 20 iterations. Time-dependent Cox regression was conducted on each of the 20 imputed datasets using the coxph function from the *survival*package^11^, and the results were pooled using Rubin’s rules to obtain final estimates, standard errors, and confidence intervals (CIs).

Univariate Cox regression was used to evaluate the association between each laboratory test, defined as a time-dependent covariate, and mortality. Variables with *p* ≤ 0.05 were included in the multivariate Cox regression model.

Multivariate Cox regression with stepwise selection was then applied, and hazard ratios (HRs) with 95% CIs were calculated. During the analysis, violations of the proportional hazards assumption were evaluated using Schoenfeld residuals, and key covariates were selected through multivariate analysis. The final selected covariates were included in a Cox regression model to evaluate their independent impact on mortality. Model fit and prediction performance were further verified through time-dependent receiver operating characteristic (ROC) analysis. The cumulative/dynamic time-dependent ROC analysis was performed using the *timeROC* package, and the area under the curve (AUC) was calculated to evaluate the prediction accuracy of each variable. The optimal cutoff value was derived based on the Youden index, and the results were organized in graphs and tables.

## Results

### Patients characteristics

A total of 440 patients diagnosed with SFTS were included in this study. The demographic and clinical characteristics of the patients are summarized in Table 1. The mean age of the patients was 66.94 ± 13.20 years, and 48.2% were male. Seventy-six patients (17.3%) died from SFTS, as recorded in the EMRs. The mean age of deceased patients was 73.28 ± 8.89 years, which was significantly higher than that of survivors (65.62 ± 13.57 years, *p* < 0.001). There was no significant difference in gender distribution between survivors and non-survivors (*p* = 0.778).

**Table 1:** Baseline characteristics of patients with SFTS.

Characteristics	Overall (*n* = 440)	Survivors (*n* = 364)	Non-survivor (*n* = 76)	*p*-value
*Demographics*				
Age (year)	66.94 (13.20)	65.62 (13.57)	73.28 (8.89)	< 0.001
Gender, n (%) Male Female	212 (48.2) 228 (51.8)	177 (48.6) 187 (51.4)	35 (46.1) 41 (53.9)	0.778
Height (cm)	162.22 (9.12)	162.14 (9.29)	162.59 (8.32)	0.714
Weight (kg)	61.50 (13.16)	61.36 (13.19)	62.19 (13.11)	0.630
*Comorbidities*				
Diabetes, n (%)	77 (17.5)	55 (15.1)	22 (28.9)	0.006
Hypertension, n (%)	164 (37.3)	127 (34.9)	37 (48.7)	0.033
*Duration*				
Onset of illness to admission (days)	4.78 (2.77)	4.79 (2.78)	4.74 (2.77)	0.877
Onset of illness to discharge or death (days)	15.40 (9.25)	16.46 (9.58)	10.29 (5.00)	< 0.001
Hospitalization (days)	11.61 (9.03)	12.67 (9.45)	6.55 (3.73)	< 0.001
*Laboratory finding*				
WBC count (×10³/µL)	2.37 (1.98)	2.36 (1.89)	2.43 (2.42)	0.780
Platelet count (×10³/µL)	73.31 (39.30)	75.82 (40.02)	60.83 (33.06)	0.003
Neutrophil count (×10³/µL)	1.45 (1.50)	1.42 (1.43)	1.57 (1.82)	0.466
Lymphocyte count (×10³/µL)	0.69 (0.59)	0.70 (0.59)	0.62 (0.54)	0.280
Monocyte count (×10³/µL)	0.16 (0.18)	0.17 (0.18)	0.12 (0.14)	0.026
Hemoglobin (g/dL)	13.52 (1.91)	13.54 (1.93)	13.38 (1.80)	0.491
Hematocrit (%)	39.75 (6.09)	39.95 (5.93)	38.76 (6.77)	0.132
Sodium (mmol/L)	135.34 (4.36)	135.30 (4.23)	135.58 (4.99)	0.615
Potassium (mmol/L)	3.95 (0.62)	3.93 (0.61)	4.03 (0.66)	0.235
AST (IU/L)	291.23 (402.11)	236.84 (306.97)	560.94 (643.12)	< 0.001
ALT (IU/L)	111.66 (138.77)	102.75 (132.77)	155.85 (159.12)	0.003
LDH (IU/L)	1048.04 (1083.16)	884.82 (769.15)	1873.88 (1827.72)	< 0.001
Total Bilirubin (mg/dL)	0.46 (0.37)	0.44 (0.34)	0.55 (0.52)	0.022
Total Protein (g/dL)	6.15 (0.73)	6.22 (0.70)	5.79 (0.79)	< 0.001
Albumin (g/dL)	3.57 (0.52)	3.63 (0.49)	3.27 (0.58)	< 0.001
BUN (mg/dL)	21.24 (13.99)	19.80 (12.51)	28.49 (18.27)	< 0.001
Creatinine (mg/dL)	1.06 (0.64)	1.01 (0.59)	1.29 (0.80)	0.001
CRP (mg/L)	66.84 (44.93)	65.14 (41.60)	75.44 (58.58)	0.089
Troponin-I (ng/mL)	4.99 (17.77)	4.40 (18.18)	8.19 (15.07)	0.117
CK-MB (ng/mL)	0.73 (4.21)	0.76 (4.68)	0.63 (1.32)	0.883
PT (sec)	8.91 (15.24)	7.47 (12.71)	14.33 (21.64)	0.003
aPTT (sec)	12.31 (1.67)	12.08 (1.13)	13.43 (2.96)	< 0.001
eGFR (mL/min/1.73 m²)	44.76 (16.09)	42.13 (10.65)	57.77 (28.05)	< 0.001
pH	7.18 (0.62)	7.15 (0.66)	7.34 (0.34)	0.024
PaCO_2_ (mmHg)	36.09 (8.09)	36.87 (7.62)	33.10 (9.17)	0.001
PaO_2_ (mmHg)	56.38 (30.64)	54.67 (30.09)	63.00 (32.06)	0.052

Data are presented as mean (standard deviation) for continuous variables and count (percentage) for categorical variables. Laboratory findings and variable index are evaluated among available data.

Laboratory results at admission showed significant differences between survivors and non-survivors. The platelet count in non-survivors was 60.83 × 10³/µL, significantly lower than that in survivors (75.82 × 10³/µL, *p* = 0.003). Non-survivors also exhibited significantly higher creatinine levels (1.29 vs. 1.01, *p* = 0.001) and lower albumin levels (3.27 g/dL vs. 3.63 g/dL, *p* < 0.001) compared to survivors. Furthermore, BUN levels were elevated in non-survivors (28.49 vs. 19.80, *p* < 0.001). Inflammatory and organ dysfunction markers, such as AST (560.94 IU/L vs. 236.84 IU/L, *p* < 0.001), ALT (155.85 IU/L vs. 102.75 IU/L, *p* = 0.003), and LDH (1873.88 IU/L vs. 884.82 IU/L, *p* < 0.001), were significantly higher in non-survivors. Coagulation-related markers, including PT (13.43 vs. 12.08, *p* < 0.001) and aPTT (57.77 s vs. 42.13 s, *p* < 0.001), were also significantly prolonged in non-survivors.

### Independent risk factors for mortality in SFTS patients

In the univariate Cox regression analysis with time-dependent covariates, several variables were significantly associated with increased in-hospital mortality (Table 2). Key variables included elevated WBC count (HR 1.181, 95% CI 1.115–1.251, *p* < 0.001), elevated neutrophil count (HR 1.066, 95% CI 1.036–1.098, *p* < 0.001), and reduced platelet count (HR 0.987, 95% CI 0.978–0.996, *p* = 0.006). Among biochemical markers, elevated potassium levels (HR 2.454, 95% CI 1.867–3.325, *p* < 0.001), elevated BUN levels (HR 1.042, 95% CI 1.033–1.050, *p* < 0.001), prolonged PT (HR 1.160, 95% CI 1.095–1.230, *p* < 0.001), and prolonged aPTT (HR 1.036, 95% CI 1.029–1.042, *p* < 0.001) emerged as predictive variables for mortality.

**Table 2:** Time-dependent Cox regression analysis of mortality risk factors in patients with severe fever with thrombocytopenia syndrome.

Variable	Univariate cox regression		Multivariate cox regression	
	Hazards ratio (95% CI)	*p*-value	Hazards ratio (95% CI)	*p*-value
WBC count	1.181 (1.115, 1.251)	< 0.001		
Platelet count	0.987 (0.978, 0.996)	0.006		
Neutrophil count	1.066 (1.036, 1.098)	< 0.001	1.063 (1.017, 1.112)	0.007
Lymphocyte count	0.959 (0.637, 1.445)	0.839		
Monocyte count	0.351 (0.086, 1.431)	0.142		
Hemoglobin	0.890 (0.768, 1.031)	0.118		
Hematocrit	0.971 (0.934, 1.01)	0.141		
Sodium	1.072 (1.009, 1.138)	0.025		
Potassium	2.454 (1.867, 3.225)	< 0.001		
AST	1.001 (0.985, 1.017)	0.950		
ALT	1.002 (1.001, 1.002)	< 0.001		
LDH	1.000 (1.000, 1.000)	< 0.001	1.000 (1.000, 1.000)	< 0.001
Total Bilirubin	1.790 (1.513, 2.118)	< 0.001	1.311 (1.111, 1.546)	0.002
Total Protein	0.359 (0.165, 0.783)	0.011		
Albumin	0.107 (0.063, 0.184)	< 0.001	0.469 (0.263, 0.835)	0.011
BUN	1.042 (1.033, 1.050)	< 0.001	1.031 (1.023, 1.040)	< 0.001
Creatinine	2.186 (1.751, 2.729)	< 0.001		
CRP	1.012 (1.006, 1.018)	< 0.001		
Troponin-I	1.069 (1.037, 1.102)	< 0.001		
CK-MB	1.160 (1.095, 1.230)	< 0.001		
PT	1.036 (1.029, 1.042)	< 0.001	1.146 (1.080, 1.215)	< 0.001
aPTT	1.016 (1.012, 1.020)	< 0.001	1.014 (1.004, 1.025)	0.008
pH	1.173 (0.805, 1.709)	0.400		
PaCO_2_	1.011 (0.959, 1.067)	0.674		
PaO_2_	1.013 (1.009, 1.018)	< 0.001		

Abbreviations: SFTS, Severe fever with thrombocytopenia syndrome; CI, confidence interval; AST, Aspartate aminotransferase; ALT, alanine aminotransferase; LDH, lactate dehydrogenase; BUN, blood urea nitrogen; CRP, C-reactive protein; CK-MB, creatine phosphokinase MB fraction; PT, prothrombin time; aPTT, activated partial thromboplastin time; eGFR, estimated glomerular filtration rate.

The results of the multivariate Cox regression analysis with time-dependent covariates, based on variables selected in the univariate analysis, are summarized in Table 2. Independent predictors for mortality were identified as neutrophil count (HR 1.063, 95% CI 1.017–1.112, *p* = 0.007), LDH (HR 1.000, 95% CI 1.000–1.000, *p* < 0.001), total bilirubin (HR 1.311, 95% CI 1.111–1.546, *p* = 0.002), albumin (HR 0.469, 95% CI 0.263–0.835, *p* = 0.011), BUN (HR 1.031, 95% CI 1.023–1.040, *p* < 0.001), PT (HR 1.146, 95% CI 1.080–1.215, *p* < 0.001), and aPTT (HR 1.014, 95% CI 1.004–1.025, *p* = 0.008).

## Time-Dependent ROC analysis and predictive performance

**Fig. 1:**
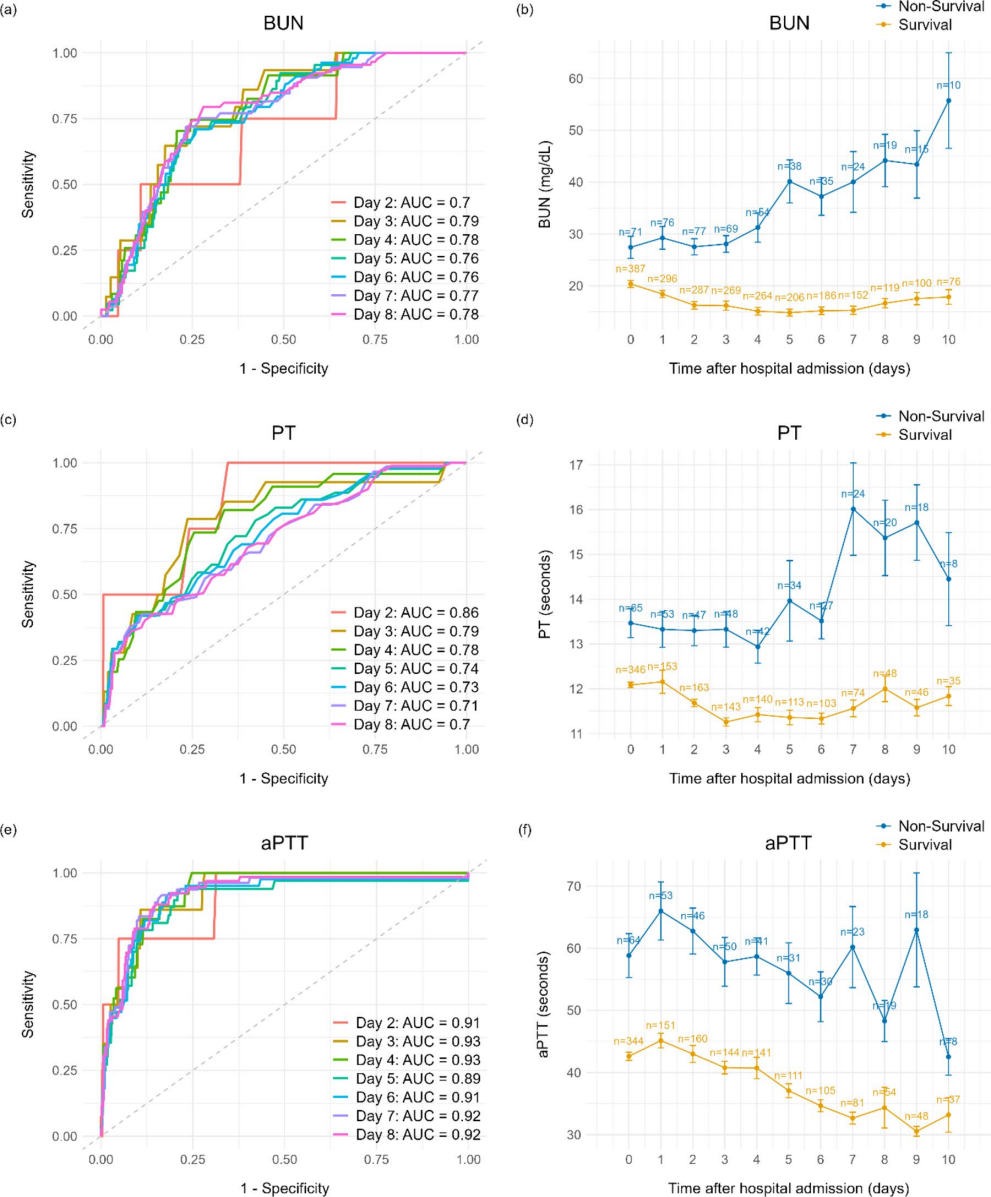
Time-Dependent Prognostic Biomarkers in SFTS: ROC Analysis and Dynamic Profile of Key Biomarkers.

Time-dependent ROC analysis was conducted to evaluate the prognostic accuracy of mortality predictors. A time-dependent ROC curve was generated to assess prediction performance over time, with AUC values calculated at each time point from day 2 to day 8 (Fig. 1 (a, c, e)). The analysis identified BUN, PT, and aPTT as key variables predicting in-hospital mortality among SFTS patients. In particular, aPTT consistently demonstrated the highest prediction accuracy, indicating its reliability in evaluating SFTS prognosis. Although PT exhibited high predictive accuracy in the early stages, its performance decreased over time, suggesting its utility is limited to early-stage monitoring. Conversely, BUN demonstrated relatively stable performance with a gradual increase in predictive power over time, indicating its reliability as a predictive variable throughout the disease course.

Moreover, the dynamic profiles of the three key mortality predictors (BUN, PT, and aPTT) identified through multivariate Cox regression and time-dependent ROC analysis were illustrated in Fig. 1 (b, d, f). The graphs depict the fluctuations in predictor levels over the course of SFTS progression. In survivors, BUN remained within the normal range, but in non-survivors, it gradually increased over time. Both PT and aPTT exhibited consistently higher levels in non-survivors. Notably, aPTT was prolonged beyond the normal range from the early stage, consistently maintaining a distinct separation between survivors and non-survivors throughout the disease course, supporting its robust predictive performance at all time points.

## Discussion

SFTS is a viral disease characterized by high mortality and rapid progression, particularly among vulnerable populations such as the elderly and immunocompromised individuals^2–4,9,16^. Most existing studies on mortality prediction in SFTS have focused on evaluating prognosis and analyzing relevant factors based on clinical outcomes measured at the time of hospital admission. Although this approach is useful for assessing a patient’s condition at a specific point in time, it fails to reflect the dynamic nature of acute infectious diseases such as SFTS, where clinical outcomes change rapidly over time.

To address these limitations, this study applied a time-dependent Cox regression model to dynamically analyze the impact of biomarker changes on mortality risk throughout hospitalization. The conventional Cox model assumes that covariates remain constant over time, limiting its ability to measure biomarker fluctuations during disease progression. In contrast, the time-dependent Cox model integrates time-varying covariates, making it more suitable for analyzing the evolving nature of acute conditions such as SFTS. Given the rapid progression of SFTS, the rate and pattern of biomarker changes are critical determinants of prognosis, necessitating an analytical method that reflects these temporal variations. By using time-dependent covariates, this study effectively measured biomarker dynamics and provided more accurate risk predictions. Thus, the time-dependent Cox model serves as an appropriate statistical method for prognostic analysis in SFTS and offers a robust basis for investigating the time-dependent characteristics of acute infectious diseases. This study utilized a time-dependent Cox regression approach to analyze all laboratory data collected during hospitalization from a cohort of 440 SFTS patients across 10 hospitals in South Korea. The analysis revealed that coagulation markers (PT and aPTT) and renal function markers (BUN) played a significant role in predicting in-hospital mortality risk.

In this study, aPTT, a key marker of the intrinsic coagulation pathway, maintained consistently high AUC values throughout the study period., indicating that coagulation dysfunction related to the intrinsic pathway plays a sustained role in mortality risk. The intrinsic pathway is critical in regulating long-term coagulation and thrombus formation, particularly following the activation of the extrinsic pathway during the early phase of infection^17^. The persistent predictive value of aPTT in our study emphasizes its potential as an important biomarker for assessing overall coagulopathy in SFTS patients. Similarly, Ye Wang et al. reported that aPTT and other coagulation-related markers were significantly prolonged in SFTS patients, identifying aPTT as an independent risk factor for SFTS-related mortality^18^. Several other studies have also shown that hemorrhagic manifestations were more frequent in deceased SFTS patients and that aPTT was significantly prolonged in the early stages of the disease^19–21^. Moreover, aPTT levels have been positively correlated with SFTSV viral load, suggesting a mechanistic link between viral pathogenesis and coagulation dysfunction^22^. Mechanistically, SFTSV has been found to cause vascular endothelial damage and activate the intrinsic coagulation system, ultimately leading to prolonged aPTT^23^. These findings are consistent with the results of the present study, indicating that prolonged aPTT is a critical risk factor for poor prognosis in SFTS patients.

PT, another marker reflecting the extrinsic coagulation pathway, was also identified as a risk factor for poor prognosis in SFTS patients. These findings are consistent with previous studies that have identified PT as an important predictor of mortality in SFTS patients^24,25^, further supporting the role of coagulation abnormalities in SFTS mortality. However, most previous studies have focused on PT values measured at the time of hospital admission, providing only a static assessment of coagulation function. The present study analyzed the temporal changes in PT throughout hospitalization, providing a more comprehensive perspective on its prognostic value. Our results showed that the AUC value of PT decreased over time, suggesting that PT is particularly useful as a prognostic marker during the initial phase of SFTS, reflecting the significant role of extrinsic coagulation dysfunction in the early pathophysiology of the disease.

BUN is a widely used serum biomarker for assessing kidney function due to its ease of measurement and clinical applicability. Renal impairment is commonly observed in the early stages of various infectious diseases, and previous studies have reported that BUN levels are significantly elevated in severe cases of SFTS and COVID-19, indicating its potential as a prognostic marker^6,26–28^. In this study, BUN exhibited a progressive increase in AUC values over time, suggesting that kidney dysfunction becomes increasingly relevant as the disease progresses. This finding indicates the increasing impact of renal impairment on SFTS prognosis in later stages and proposes the need for continuous monitoring of kidney function in critically ill patients^29–31^.

These results suggest that the management strategy for SFTS patients should be adjusted according to the disease progression. In the early stages, it is critical to rapidly assess and correct coagulation dysfunction by monitoring aPTT and PT, whereas in the later stages, continuous monitoring and management of kidney function markers, such as BUN, are essential to improving prognosis.

Although this stepwise approach is an important sign that the patient’s condition is progressing to severity, it cannot be confirmed through fixed covariate analysis. However, the time-dependent covariate model effectively evaluates the prognosis of SFTS patients by reflecting changes in clinical outcomes over time (and mortality). It is anticipated that such an approach can contribute to reducing in-hospital mortality risk. In particular, it emphasizes the need to flexibly adjust management and treatment strategies as the patient’s condition evolves. Continuous monitoring of the patient’s condition and timely implementation of appropriate interventions through dynamic clinical outcome analysis are essential for improving the survival rate of SFTS patients.

The clinical decision support system (CDSS), which is integrated with EMR systems, is essential for the practical implementation of time-dependent analyses in clinical settings. Currently, most healthcare institutions rely on clinicians to manually review patient data and make decisions accordingly; however, real-time risk prediction could be achieved through automated data collection and analysis using CDSS. CDSS embedded with the time-dependent algorithm could enhance clinical utility by providing automated alerts when specific biomarker fluctuations reach critical thresholds.

### Limitations

This study has several limitations. First, missing laboratory data were inevitable due to the retrospective design and variability in clinical practice across institutions. To address this limitation, multiple imputations were employed; however, imputation may introduce bias depending on the underlying data distribution. Second, as a retrospective multicenter observational analysis, it inherently limits the ability to measure the impact of interventions on biomarker dynamics and patient prognosis. In particular, patients with greater disease severity were more likely to receive intensive treatment, introducing potential selection bias that may have influenced the results. Furthermore, treatment strategies varied across institutions, and clinical decisions were made at the discretion of individual physicians, further complicating the interpretation of findings. These factors present challenges in accurately determining causal relationships between treatment interventions and biomarker changes. Third, the generalizability of our findings requires careful consideration. This study was conducted using multicenter data from South Korea, and our findings are consistent with previous studies conducted in China, which identified prognostic biomarkers for SFTS-related mortality. However, due to differences in environmental, genetic, and healthcare system factors, direct generalization of our results to other endemic regions, such as China and Japan, remains challenging. To improve generalizability, external validation using independent datasets is necessary, and further multinational studies are required to confirm these findings across diverse populations.

## Conclusions

In summary, our study demonstrates that using a time-dependent Cox model may significantly improve the prognostic evaluation of SFTS by measuring dynamic changes in key biomarkers. Notably, coagulation markers such as PT and aPTT, along with renal function indicator BUN, provide robust predictive power throughout the disease course. These findings support the integration of dynamic biomarker monitoring into clinical decision-making to facilitate timely interventions. Future prospective studies are needed to further validate these results and enhance risk stratification in the treatment of SFTS.

## Electronic supplementary material

Below is the link to the electronic supplementary material.

Supplementary Material 1

## Data Availability

The data sets generated and analyzed in the study are available from the corresponding author upon reasonable request.

## Abbreviations

SFTS: Severe fever with thrombocytopenia syndrome
AST: Aspartate aminotransferase
ALT: alanine aminotransferase
LDH: lactate dehydrogenase
BUN: blood urea nitrogen
CRP: C-reactive protein
CK-MB: creatine phosphokinase MB fraction
PT: prothrombin time
aPTT: activated partial thromboplastin time
eGFR: estimated glomerular filtration rate
