# Supplementary material for: Dynamic biomarkers and Cox regression with time-dependent covariate for mortality prediction in severe fever with thrombocytopenia syndrome
Source: Sci Rep. 2025 Mar 18;15:9293. doi: 10.1038/s41598-025-94416-0 (PMC11920429; doi:10.1038/s41598-025-94416-0)
Supplement: Supplementary file 1 — Supplementary Material 1 [file 41598_2025_94416_MOESM1_ESM.docx]

Supplementary Table 1. List of IRBs and IRB approval numbers

| Name of IRBs | Contact number | IRB approval number |
| --- | --- | --- |
| Chonnam National University Hospital | +82-62-220-5257 | CNUH-2023-213 |
| Chonnam National University Hwasun Hospital | +82-61-379-7598 | CNUH-2024-150 |
| Gyeongsang National University Hospital | +82-55-750-9252 | GNUH 2024-08-020 |
| Jeju National University Hospital | +82-64-717-1503 | JEJUNUH 2024-08-002 |
| Jeonbuk National University Hospital | +82-63-259-3339 | CUH 2022-01-067 |
| Keimyung University Dongsan Hospital | +82-53-258-6693 | DSMC 2024-08-008 |
| Kyungpook National University Chilgok Hospital | +82-53-200-2162 | DGIRB 2022-05-004 |
| Kyungpook National University Hospital | +82-53-200-5430 | DGIRB 2023-07-001 |
| Wonkwang University Hospital | +82-63-859-2234 | WKUH 2024-08-005 |
| Konkuk University Medical Center | +82-2-2030-6516 | KUMC 2024-09-001 |

IRB, Institutional Review Board
